# The Interspinous Spacer: A New Posterior Dynamic Stabilization Concept for Prevention of Adjacent Segment Disease

**DOI:** 10.1155/2013/637362

**Published:** 2013-04-10

**Authors:** Antoine Nachanakian, Antonios El Helou, Moussa Alaywan

**Affiliations:** Department of Neurosurgery, Saint George Hospital University Medical Center and Balamand University, Youssef Sursock Street, Rmeil, Beirut 11 00 2807, P.O. Box 166378, Lebanon

## Abstract

*Introduction*. Posterior Dynamic stabilization using the interspinous spacer device is a known to be used as an alternative to rigid fusion in neurogenic claudication patients in the absence of macro instability. Actually, it plays an important in the management of adjacent segment disease in previously fused lumbar spine. *Materials and Method*. We report our experience with posterior dynamic stabilization using an interspinous spacer. 134 cases performed in our institution between September 2008 and August 2012 with different lumbar spine pathologies. The ages of our patients were between 40 and 72 years, with a mean age of 57 years. After almost 4 years of follow up in our patient and comparing their outcome to our previous serious we found that in some case the interspinous distracter has an important role not only in the treatment of adjacent segment disease but also in its prevention. *Results and Discussion*. Clinical improvement was noted in ISD-treated patients, with high satisfaction rate. At first, radicular pain improves with more than 3/10 reduction of the mean score on visual analog scale (VAS). In addition, disability score as well as disc height and lordotic angle showed major improvement at 3 to 6 months post operatively. And, no adjacent segment disease was reported in the patient operated with interspinous spacer. *Conclusion*. The interspinous spacer is safe and efficient modality to be used not only as a treatment of adjacent segment disease but also as a preventive measure in patients necessitating rigid fusion.

## 1. Introduction 

Spinal disorders are among the most common health complaints affecting a large portion of the population in developed and developing countries [1]. Spinal disorders can be treated medically at first and the majority of patients will respond to the latter, whereas others will need surgical treatment for their spinal disease. And though, degenerative disease of the spinal cord became a serious problem with the aging of the population and its management is in continuous evolution. 

Spinal stenosis manifested by back or radicular pain and nonresponding to conservative management or evolution to neurogenic claudication necessitates surgical procedure. The management of this pathology changed over time and in case decompressive surgery was not sufficient or the spinal segments degenerated later on, a rigid fusion was used. Rigid fusion was efficient and provided better outcome compared to decompression alone, but it could not resolve the problem of disc degeneration without evident radicular compression [2]. And, overtime,with millions of segmental fusion done, a new pathology known as adjacent segment disease was described. From this evidence for adjacent-segment degeneration emerged the concept of dynamic or nonfusion stabilization of the lumbar spine [3]. The dynamic stabilization hardware functions as shock absorbent at the level above a fused segment and reduces the pressure leading to further degeneration in the spinal cord [4].

 Posterior dynamic stabilization was born from the need of normalization of the intersegmental motion [5] and in contrast to the traditional fusion surgery it does not eliminate the mobility of the fused segment [6]. While both procedures treat the microinstability, posterior dynamic stabilization does it in a more physiological manner. By restoring normal motion, mobility is theoretically preserved rather than eliminated, and the forces acting above and below the construct are altered to a lesser extent, reducing the potential undesirable effects of fusion [7]. 

Interspinous process spacers have been introduced as a possible alternative to spinal decompression and fusion for the treatment of neurogenic intermittent claudication (NIC) and discogenic lower back pain [8]. The interspinous devices work as a shock absorbent device. In addition to intervertebral height restoration, it improves central canal and foraminal stenosis. Interspinous Distracter (ISD) is designed to stabilize the motion segment after neural elements decompression in lumbar stenosis, tolerating flexion and extension in this segment thus preserving the adjacent segment from deterioration [5–8].

## 2. Methods 

Our experience is based on 134 cases performed between September 2008 and August 2012 with different lumbar spine pathologies (Table 1). The ages of our patients were between 40 and 72 years, with a mean age of 57 years. All patients were treated with Interspinous Distracter (ISD).

### 2.1. Inclusion Criteria

**Figure 1:**
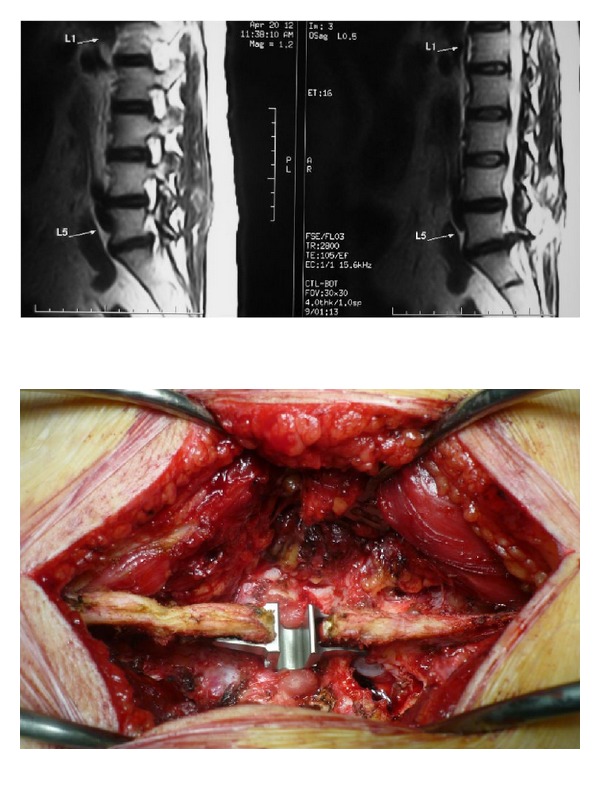
On the left, sagittal T2W image MRI of Lumbosacral spine showing extruded L5-S1 disc with degenerated L4-L5 disc. On the right, preoperative view of the same patient showing left L5-S1 laminotomy for disc excision and L4-L5 interspinous distractor.

At the beginning of our usage of ISD, patients were eligible for enrolment if they had the following:

- degenerative disk disease and subsequent bilateral foraminal stenosis (Figure 1),
- foraminal-canalar stenosis, due to ligamentum flavum hypertrophy, declared symptoms consisting of neurogenic claudication,
- suspended vertebra shown on X-ray which is due to facet degenerative disease,
- facet joint syndrome.

**Figure 2:**
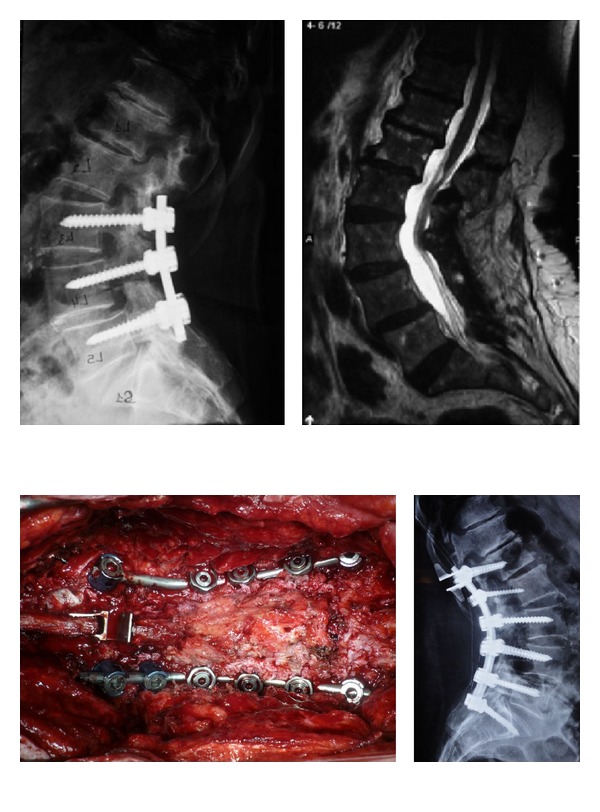
Above preoperative spine X-ray with previous instrumented level on the right. On the left, degenerated segments at L1-L2 and L2-L3 levels. Below preoperative view showing the extended fusion from L1 to S1 with Th12-L1 interspinous spacer.

Then with the development of the techniques and the follow-up results, two indications were added to the above mentioned criteria:

- adjacent segment syndrome (Figure 2) which refers to degenerative changes that occur in the mobile segment next to spinal fusion,
- degenerated disc at a level superior to the one necessitating posterior rigid fusion.

After several procedures with successful results in management of adjacent segment syndrome and to avoid later hospitalization and added surgical procedure in previously operated patients with spinal fusion, we started to use ISD as prevention to avoid adjacent segment disease.

### 2.2. Exclusion Criteria

Patients were excluded in cases of more than 2 adjacent levels disease, in the absence of the spinous processes due to previous surgery or fractures, in the presence of spondylolysthesis, and, when severe osteoporosis exists in the lumbar region (*T*  score < −2.5 in the lumbar region).

### 2.3. Preoperative Evaluation

The patients completed the visual analogue scale (VAS) for pain and Oswestry disability index (ODI).

At first, paraclinical evaluation included plain lumbar film, lumbar MRI or CT, and osteodensitometry. Then MRI along with dynamic lumbar X-ray was used and osteodensitometry was done in postmenopause female patients.

 The global and segmental lordotic angles (stabilized segments, above and below adjacent segments) were measured using Cobb's method on lateral neutral position lumbosacral spine X-ray.

The segmental lordotic angles (stabilized segments and adjacent segments) were measured from between the upper end plates of the corresponding segments.

### 2.4. Operative Procedure

#### 2.4.1. Preparation

The procedure is done under general anesthesia. All patients were operated in a prone position, avoiding hyperlordosis for a better interspinous distraction. 

#### 2.4.2. Product Used

Different interspinous spacers' types are used in our institution. 

#### 2.4.3. The Instrument Used

A set of lumbar laminectomy is used. In addition, a set of interspinous spacer measurer is utilized to define the depth and width of the spacer to be used. 

#### 2.4.4. Surgical Note

The level of the procedure is localized under fluoroscopy after positioning. Surgical exposure is done similar to any lumbar laminectomy procedure. For the insertion of the ISD the interspinous ligament as well as the ligamentum flavum was resected.

After ISD insertion, the depth between it and the dural sac is assessed by 3 mm hook.

**Figure 3:**
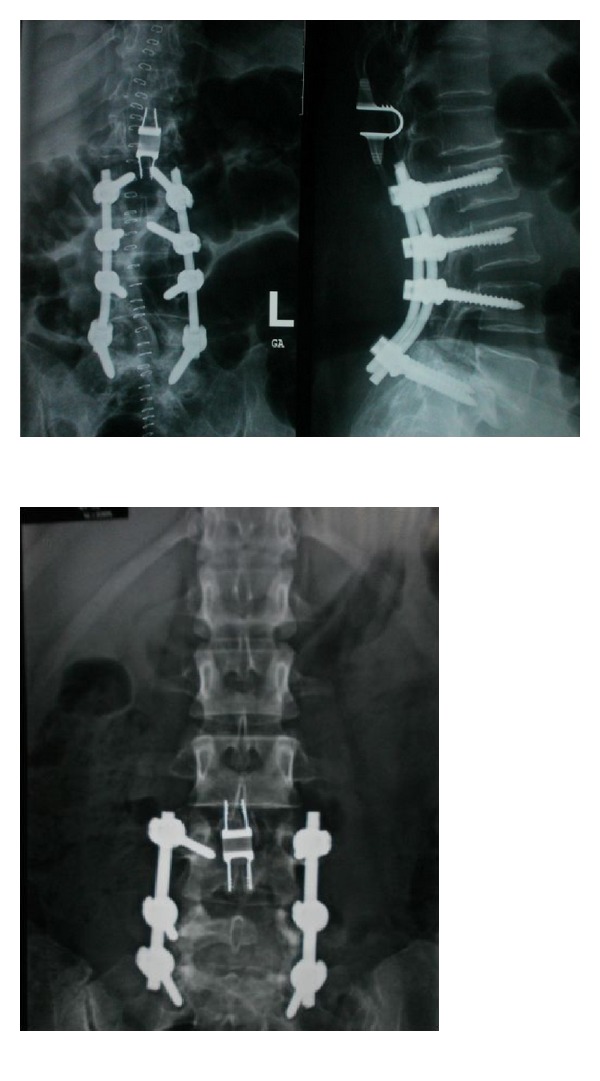
Postoperative spine X-ray, on the right, showing multiple level fusions with ISD 1 level skipping the adjacent segment. On the left, ISD used at the level adjacent to the rigid fusion.

In cases where the disc is protruded/herniated, medial discectomy was not done. In cases where degenerative or congenital spondylolysthesis is present, rigid fusion of the spondylotic level was done. The insertion of ISD at the level above was done in patients older than 55 years. In patients younger than 55 years, the decision was made for each case separately. If the level adjacent to the fusion is not degenerated, this level is spared and the ISD is inserted at the level above; whereas if the disc at the adjacent level is degenerated, ISD is used at the latter mentioned level (Figure 3). 

Regular closure of layers and placing of deep hemovac drain ended the surgery.

### 2.5. Follow-Up Evaluation

#### 2.5.1. Immediate Postoperative Care

The patient is out of bed the day after surgery and discharged on day 3 after surgery or on day 2 when drain was not inserted. All patients wear a lumbar brace, for a period of one month during their daily activities. 

#### 2.5.2. Late Postoperative Evaluation

The following data were collected: VAS, ODI, pain medication, complications, and patient satisfaction. 

Control lumbosacral X-ray is done in 2 views to evaluate the created distraction. 

The plain radiographs (anteroposterior and lateral standing in neutral position) are obtained at day 1, day 90, and day 180 postoperatively. Disc height and Cobb's angle are measured and compared to the preoperative values. 

### 2.6. Complications

In general, materials are well tolerated. The rate of complications is between 1% and 10% in all series. Two sets of complications exist: the early and the delayed.

Early complications include device dislocation/malposition, spinous process fractures, erosion of the spinous process, infection, hematoma, and neurological sequelae.

One case of migration was observed in one series [4]. There were no broken or permanently deformed implants in all series.

In our series, no cases of fracture of the superior spinous process occurred. In our experience, we do osteodensitometry for all patients to assess bone density preoperatively. During operation, we avoid bone erosions of the adjacent spinous processes. 

We had one case of recurrent neurological symptoms, and ISD was removed. Microsurgical decompression and posterolateral fusion were done. To avoid this type of complications, a complete posterior decompression through ligamentum flavum excision and discectomy in the presence of herniated disc should be done.

Selection of patients without spondylolysthesis is mandatory to avoid posterolateral fusion later on. And in the presence of spondylotic segment, rigid fusion with insertion of ISD at the superior adjacent level protects from recurrence of neurological symptoms as well as from later adjacent segment disease. 

### 2.7. Statistical Analysis

The clinical and radiologic results were analyzed using *t*-test; a *P* value of less than 0.05 is considered statistically significant. All analyses were carried out using SPSS Ver. 16.00 (IL, Chicago, Inc.).

## 3. Results

### 3.1. Pain Assessment

Overall improvement was noted in ISD-treated patients, with considerable satisfaction in 89% of patients on average. 

The patients at first reported an improvement of their radicular pain with a mean reduction of 3.4/10 on visual analog scale (VAS) (scale for 0: absent pain to 10: severe intolerable pain necessitating intravenous treatment).

In the preoperative period, radicular pain had a mean score of 8.6/10 on VAS (5–10). Whereas in the immediate post-op period, the pain mean score was 4.3/10 on VAS (1–7).

**Figure 4:**
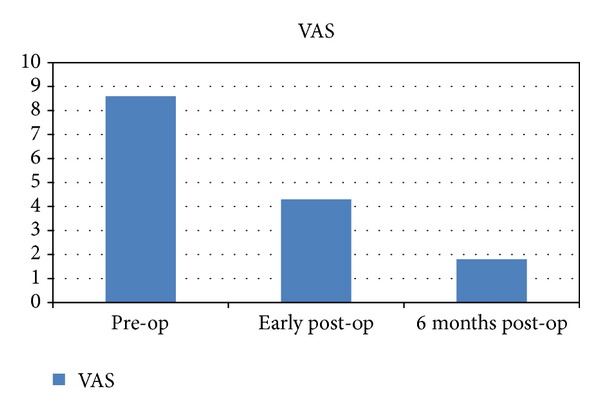
Comparative chart of mean VAS from preoperative period, early postoperative, and at 6-month follow up.

Patients achieved maximum improvement after an average period of 6 months, with a mean score of 1.8/10 on VAS (0–5), and up to 83% of patients were pain free (Figure 4).

### 3.2. Disability Assessment

**Figure 5:**
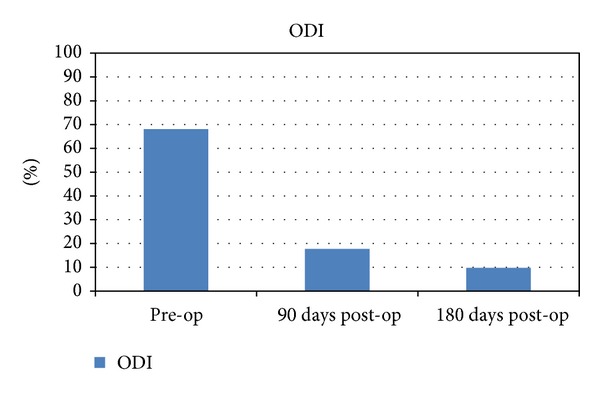
Oswestry low back disability score comparing pre-op evaluation to 3 months and 6 months post-op evaluation.

The Oswestry low back disability questionnaire score (ODI) improved from a mean of 68.1% in the pre-op period (23%–91%) to 17.8% at 3 months (0%–52%) and <10% at a 6-month followup (*P* < 0.05) (Figure 5).

### 3.3. Disc Height

The preoperative disc height was measured by MRI, with a mean of 1.2 cm (0.4–1.6 cm) and intervertebral space on lateral X-ray view measured manually had a mean of 1.3 cm (0.7–1.6 cm). Whereas, in post-op evaluation only spine X-ray was done (due to the elevated cost of MRI) and the mean measured intervertebral space was 1.75 cm (1.2–2.4 cm). 

**Figure 6:**
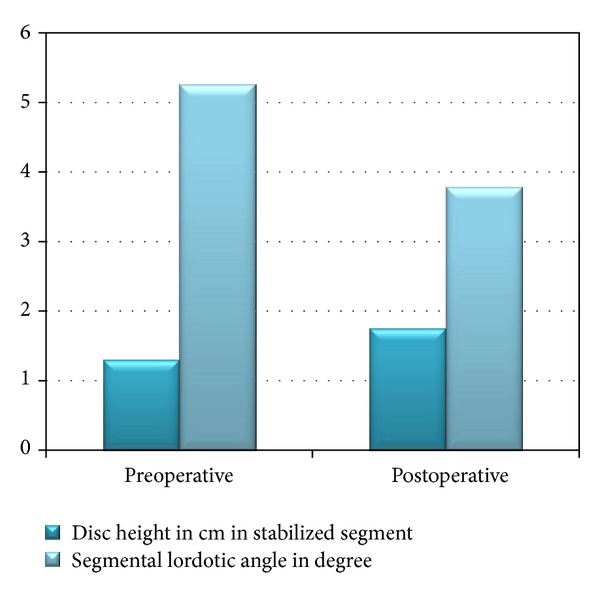
Comparative chart between preoperative and postoperative radiological changes.

Radiologic changes, on lateral views in neutral position in lumbosacral spine X-ray, in the disk height of the stabilized segment, were increased significantly from preoperative to immediate postoperative evaluation (*P* < 0.05). This increase persisted at 3-month followup (*P* < 0.05) (Figure 6). 

### 3.4. Segmental Lordotic Angles

The range of motion measured by the segmental lordotic angle in stabilized segment decreased postoperatively (3.78 ± 3.1°) compared to the preoperative measured values (5.26 ± 3.68°). This change was not statistically significant (*P* = 0.4). 

Although adjacent segment ROM showed a decrease on post-op X-ray, there was no statistical significance (Figure 6).

### 3.5. Operative Characteristics

The prominent characteristic of this surgery is a low level of postoperative pain. And so, the decompression is done by removal of ligamentum flavum and the reestablishment of the dynamics of the spine plays a major role in the resolution of back pain. Restoration of the height of the intervertebral disc relieves the pressure on the sinuvertebral nerve which plays a major role in decreasing paraspinal muscles spasm despite the back pain.

In addition, the amount of blood loss with ISD procedure (49.2 cc ± 24.8) compared to rigid stabilization (184.3 cc ± 67.8) was found to be reduced (*P* < 0.005).

## 4. Discussion

Rigid spinal fusion is a mandatory procedure for the management of lumbar instability although it could be associated with different types of complications such as device failure, osteoporosis, and spinal deformity by changing the spinal mechanical activities, leading to adjacent segment disease [9]. The fusion technique shifts the center of rotation of vertebral body over the disc leading to an increase in the stress on the facets and/or disc of the adjacent mobile segment. The increase of stress induces several changes in the mobility of the adjacent segment and elevation of intradiscal pressure [10]. And so, it can lead to disc degeneration which precedes the facet degeneration [11]. 

Some authors do not agree with the theory of adjacent segment degeneration and in a prospective study conducted in Spain, disc degeneration post lumbar fusion appeared homogeneously at several levels cephalad to fusion and seemed to be determined more by individual characteristics than by fusion itself [12]. 

In our experience, after a long followup period, we have remarked that adjacent segment disease is a serious problem that causes refractory pain to medical treatment, which necessitated long segment fusion which leads to limitation of back motion and spinal deformities [13]. In addition, the refractory pain to medical treatment has high cost both on individual and national levels.

To avoid these adverse effects, the achievement of ideal mobility is important. Thus, dynamic stabilization devices would appear to represent a notable technological advantage. 

Posterior dynamic stabilization is done to decrease and/or avoid the harmful effects of rigid fusion, like listhesis, instability, hypertrophic facet joint arthritis, herniated nucleus pulposus, and stenosis.

Several studies comparing interspinal distractor or dynamic pedicular system to posterior lumbar interbody fusion (PLIF) were conducted and showed promising results [14, 15]. We did not try dynamic pedicular system since we had satisfactory results with the interspinous spacer.

 The interspinous dynamic stabilization system, with preservation of the disc and facet, creates a favorable environment in the motion segment by reducing the loading on these joints and allowing more normal motion. 

The clinical outcomes of patients in our study improved significantly during the follow-up period, not only at 3 and 6 months, but also in the early post-op period. 

The system increased the distraction posteriorly and improved the anterior disc space height and articular process pressure which decreased the stenosis, liberated the nerve roots and the foramina [14], and reduced neural pain transmission via the dorsal root ganglia despite decreased overall painful stimuli and transmission [16]. 

The followup with serial X-rays showed no evidence of osteophytes at articular facets level as noted in rigid fixation. We conclude that ISD not only decreases the load on the facets, but also impairs osteophytes formation. Decreasing the load in addition to the impairment of osteophytes formation is an additional proof that posterior dynamic stabilization is an effective method to treat and to prevent adjacent segment disease. It also shows that this type of fusion not only preserves normal motion, but it prevents further degenerative process by maintaining patient's own lumbar kinematics and reducinginstability.

In late postoperative X-ray followup of the patients examined, a mineralization of the spinous process in contact with the implant was found, in particular at its base which appears to absorb high stresses due to lordosis, and this finding was described 10 years ago [17]. 

Our results concerning disc height and segmental lordotic angles correlate with other studies done in China and Turkey [15, 18]. This means that the ISD is useful regardless of ethnic origin. 

Rigid stabilization was found to decrease fiber strain of the intervertebral disc and transfer the load to the rod, changing all the biomechanics of the spinal cord; whereas ISD keeps the natural fiber strain in a physiological manner which was proved by the pre- and postoperative disc height [16].

The ISD system slightly limits the bulging of the disc at the lateral and posterolateral site. This could be due to the decompression effect at the posterior elements by the implant [19]. Compared to rigid stabilization surgery, ISD insertion is associated with less blood loss and shorter surgical time and hospital stay. These criteria have a high impact on postoperative pain, recovery period, and the overall quality of life [20].

## 5. Conclusion

Interspinous spacer insertion after excision of ligamentum flavum showed excellent results in terms of pain control, motion preservation, and prevention of adjacent segment degeneration in previously stabilized lumbar spine segments. It provides restoration of disc height, reduction of vertebral slip and leads to physiological condition concerning disc bulging. We highly recommend its use in treatment as well as in prevention of adjacent segment disease specifically in young patients where spinal fusion for early degenerative disease is needed.

## Figures and Tables

**Table 1:** Number of cases in correlation with disease and sex.

Number of cases	Pathology	Male/female ratio
36	Biforaminal stenosis	24/12
15	Ligamentum flavum hypertrophy	8/7
6	Suspended vertebrae	4/2
3	Facet syndrome	0/3
47	Adjacent syndrome	28/19
27	Adjacent syndrome prevention	12/15
